# STGATN: A novel spatiotemporal graph attention network for predicting pollutant concentrations at multiple stations

**DOI:** 10.1371/journal.pone.0328532

**Published:** 2025-07-30

**Authors:** Huazhen Xu, Wei Song, Lanmei Qian, Xiangxiang Mei, Guojian Zou

**Affiliations:** 1 College of Yongyou Digital and Intelligence, Nantong Institute of Technology, Nantong, People’s Republic of China; 2 The Key Laboratory of Road and Traffic Engineering, Ministry of Education, Tongji University, Shanghai, People’s Republic of China; 3 College of Transportation Engineering, Tongji University, Shanghai, People’s Republic of China; 4 Department of Geography, University of Zurich, Zurich, Switzerland; Newcastle University, UNITED KINGDOM OF GREAT BRITAIN AND NORTHERN IRELAND

## Abstract

Accurately predicting air pollutant concentrations can reduce health risks and provide crucial references for environmental governance. In pollution prediction tasks, three key factors are essential: (1) dynamic dependencies among global monitoring stations should be considered in spatial feature extraction due to the diffusion properties of air pollutants; (2) precise temporal correlation modeling is critical because pollutant concentrations change dynamically and periodically; (3) it is vital to avoid propagation of long-term prediction errors across spatiotemporal dimensions. To address these challenges, we propose STGATN, a novel spatiotemporal graph attention network with an encoder-decoder architecture. Both the encoder and decoder incorporate a spatiotemporal embedding mechanism, a spatiotemporal graph attention block, a gated temporal convolutional network, and a fusion gate. Specifically, the spatiotemporal graph attention module is designed to use temporal and graph attention networks to extract dynamic spatiotemporal correlations. The gated temporal convolutional network is constructed to capture the long-term temporal causal relationships. The fusion gate adaptively fuses the spatiotemporal correlations and temporal causal relationships. In addition, a spatiotemporal embedding mechanism, including positional and temporal information, is added to account for pollutants’ periodicity and station-specific properties. Moreover, this paper proposes a transformer attention that establishes direct dependencies between future and historical time steps to avoid prediction error accumulation in the dynamic decoding process. The experimental results show that the proposed prediction model significantly outperforms the latest baseline methods on the air pollution dataset from actual monitoring stations in Beijing City.

## 1 Introduction

Air pollution has become a pervasive issue globally, exacerbated by rapid industrialization in both developed and developing countries. The World Health Organization (WHO) estimates that approximately 7 million people die annually due to air pollution, posing a significant threat to human health and safety [1]. Studies show that individuals exposed to polluted air are at higher risk for respiratory and cardiovascular diseases [2, 3]. Accurate prediction of air pollutant concentrations is essential for the development and implementation of preventive measures, enhancing public health, and managing environmental impact [4]. Research indicates that air pollutant concentrations are influenced by meteorological factors and topography, including wind direction, and spread spatially over time, a phenomenon referred to as spatial dependence [5, 6]. The ongoing dynamic changes in air pollutant content at specific locations over time are referred to as temporal correlation [7, 8]. Therefore, accurately extracting complex spatiotemporal correlations is vital for precise pollutant concentration prediction.

Early air pollutant prediction studies predominantly relied on traditional numerical models. These models leveraged historical air pollution data to estimate future pollution levels, yielding relatively accurate forecasts [9]. Their strength lies in effectively modeling small datasets using mathematical and physical formulas [10]. However, their inability to capture nonlinear features in time-series data constrained further accuracy improvements. Advancements in machine learning have significantly advanced pollutant concentration prediction, with methods such as support vector regression (SVR) [11] demonstrating progress. Machine learning techniques outperform traditional numerical models in capturing nonlinear relationships between variables. However, these methods face challenges in processing the non-Euclidean spatial structures of large-scale air pollution data, hindering their ability to extract deep, complex spatiotemporal features accurately [12].

In recent years, deep learning has rapidly advanced, exhibiting exceptional performance in spatiotemporal prediction and finding widespread applications in fields such as natural language processing [13] and time series forecasting [14]. These advancements have also drawn considerable interest in air pollutant concentration prediction [5, 15]. Deep learning models employ an end-to-end architecture with multiple layers and activation functions [16], enabling automated processing from input to output. Research shows that deep learning models, due to their ability to process large datasets and capture spatiotemporal correlations, outperform traditional models in predictive accuracy [17, 18]. Multilayer perceptrons (MLPs) struggle to model temporal evolution, leading to their gradual replacement by recurrent neural networks (RNNs) [19]. Since spatial dependence is crucial in pollutant concentration prediction, convolutional neural networks (CNNs) have been widely used to extract local spatial features in early prediction models [17]. However, local features alone struggle to capture pollutant diffusion patterns and similarities in concentrations across global monitoring stations. Graph neural networks (GNNs) effectively process graph-structured data, modeling global dependencies through node and edge relationships [20]. Due to their ability to model spatial dependencies, GNNs serve as a superior alternative to CNNs in pollutant prediction tasks.

Current research on air pollutant concentration prediction can be categorized into single-station and multi-station forecasting. Historically, most studies have focused on leveraging past pollutant concentration data to predict specific pollutants (e.g., PM2.5) at individual monitoring stations. However, limited research has explored long-term pollutant concentration forecasting across multiple stations. Existing deep learning models face several challenges in air pollution prediction: (1) **Insufficient Extraction of Spatial Semantic Information** CNN-based models primarily capture local spatial features, making it difficult to model dynamic spatial dependencies between monitoring stations and extract global correlations. Research indicates that GNN-based models outperform CNNs in handling non-Euclidean spatial structures [21]. Research indicates that GNN-based models outperform CNNs in handling non-Euclidean spatial structures [21]. GNNs can be broadly classified into Graph Convolutional Networks (GCNs), Adaptive Graph Convolutional Networks (AdapGCNs) [22], and Graph Attention Networks (GATs) [23]. However, GCNs rely heavily on predefined spatial topology maps, limiting adaptability. AdapGCNs, while flexible, fail to update inter-station dependencies during inference, making them ineffective in capturing dynamic spatial relationships. (2) **Neglect of Dynamic Evolution and Causal Relationships** Pollutant concentrations exhibit periodicity, such as recurring trends on the same day across consecutive weeks. Moreover, concentration changes follow causal relationships, where past fluctuations influence current levels. However, existing models often emphasize methodological advancements while overlooking these inherent temporal patterns and dependencies. (3) **Long-Term Prediction Error Accumulation** Dynamic decoding approaches, such as those using RNN- or Transformer-based decoders [24], suffer from error propagation across temporal and spatial dimensions. This accumulation leads to reduced predictive accuracy and prolonged inference times, significantly impacting long-term forecasts. Addressing these challenges remains a critical direction for advancing air pollution prediction methodologies.

Therefore, this paper introduces a novel Spatiotemporal Graph Attention Network (STGATN) for predicting multi-station pollutant concentrations. The main contributions are as follows:

1. We propose a novel STGATN architecture to extract spatiotemporal correlations across multiple monitoring stations, enabling accurate long-term pollutant concentration forecasting. To the best of our knowledge, this study is the first to introduce a spatiotemporal graph attention block (ST-Block) that jointly models spatiotemporal dependencies in pollutant concentrations across multiple monitoring stations. This approach enables dynamic feature extraction without reliance on prior knowledge, while maintaining low computational cost.
2. Modeling temporal correlations, including dynamic evolution patterns and causal relationships, is essential for accurate pollutant concentration forecasting. A gated temporal convolutional network (GTCN) is designed to capture pollutant concentration trends while emphasizing global evolution and causal relationships. Moreover, timestamps serve as key indicators of pollutant concentration periodicity. For instance, poor air quality at 11 a.m. on Fridays may recur in consecutive weeks, reflecting temporal patterns that help prevent feature extraction biases.
3. To mitigate long-term prediction error accumulation, a specialized transformer attention (TransAtt) is designed to bridge historical and future spatiotemporal representations. This allows multi-time-step, multi-station pollutant representations to be generated in a single step instead of through dynamic decoding. This approach prevents error propagation across temporal and spatial dimensions, significantly enhancing inference speed.
4. In this study, PM2.5 is chosen as the target variable for experimental validation. Experimental results show that the proposed model surpasses baseline models in RMSE, MAE, and IA metrics.

The remainder of this paper is organized as follows: Sect 2 summarizes related research, Sect 3 defines the relevant problem, Sect 4 describes the proposed pollutant concentration prediction network in detail, Sect 5 presents the prediction results and experimental analysis, and Sect 6 concludes the paper while discussing future research directions.

## 2 Related work

Based on previous studies, air pollutant concentration prediction methods can be categorized into two main types: non-deep learning methods and deep learning methods. Non-deep learning methods are further divided into deterministic and statistical approaches.

### 2.1 The deterministic method

Deterministic methods utilize meteorological principles and complex mathematical equations to simulate pollutant emission, transformation, diffusion, and disappearance processes while considering atmospheric physical and chemical reactions [25, 26]. For instance, Chemical Transport Models (CTMs) describe the chemical transformation processes of atmospheric pollutants, focusing on establishing mathematical methods for pollutant emission, diffusion, and transformation [26]. The Weather Research and Forecasting (WRF) model is applied in atmospheric pollution prediction studies, including WRF-Chem and WRF/Chem-MADRID models [27, 28]. These models leverage well-established foundational theories to provide valuable insights for air pollution prediction. However, deterministic methods are constrained by prior knowledge, various limitations, and limited data, making them difficult to generalize in practical scenarios. These methods also struggle to establish nonlinear relationships between independent and dependent variables (target pollutants) [9, 15], challenging further improvements in pollutant concentration prediction accuracy. To address these issues, researchers have begun exploring applying statistical methods in pollutant prediction.

### 2.2 Statistical method

Compared to deterministic methods, statistical methods better reveal the nonlinear relationships between variables. These methods systematically utilize mathematical knowledge, including statistics, probability, and stochastic processes, and can be divided into two subclasses: early statistical methods and machine learning methods. Early statistical methods, such as the Autoregressive Integrated Moving Average (ARIMA) model and its variant, Seasonal ARIMA (SARIMA), have shown efficacy in specific contexts. For example, Ni *et al*. demonstrated that the ARIMA model could accurately predict PM2.5 concentrations for the next hour using the Beijing dataset [29]. However, these methods are constrained by assumptions of time series stability and data completeness, limiting their ability to fully capture nonlinear data correlations. With the rise of artificial intelligence, researchers have increasingly adopted machine learning methods to achieve better prediction results. The primary advantage of traditional machine learning methods over early statistical methods is their ability to effectively handle nonlinear problems, thereby improving prediction accuracy. Common machine learning models include SVR [11], Random Forest (RF) [30], Hidden Markov Models (HMM) [31], and other foundational models. These methods are primarily designed to capture shallow nonlinear features of input data and generally perform well on small-scale datasets. However, they struggle to leverage large-scale air pollution data and have limited capacity to extract deep and complex spatiotemporal features, making them challenging to apply widely in real-world scenarios.

### 2.3 Deep learning method

In recent years, deep learning methods have exhibited exceptional predictive performance in regression problems, resulting in the development of various spatiotemporal network architectures aimed at improving air pollutant concentration predictions. As pollutant prediction is a classic time series forecasting problem, effectively extracting temporal correlations is crucial. RNNs have demonstrated significant advantages in temporal feature extraction [32], with variants such as Gated Recurrent Units (GRUs) and Long Short-Term Memory (LSTM) networks widely used as core components in pollutant prediction tasks. For example, Zhang *et al*. proposed an end-to-end model based on LSTM that achieved precise single-station pollutant concentration predictions [4]. However, single time series prediction models can easily overlook the spatial dependencies of pollutants. To address this, some studies have combined CNNs and RNNs to achieve synchronous extraction of temporal and spatial features. A common spatiotemporal architecture is the CNN-LSTM prediction model [17]. In this model, the spatial module uses a CNN to extract local spatial dependency features among multiple stations, while the temporal module employs an LSTM to capture temporal correlations. For instance, Zhang *et al*. introduced a spatiotemporal pollutant concentration prediction model named RCL-Learning [33]. Specifically, they used a multi-layer residual network to extract spatial features of pollutants from multiple stations and employed a convolutional LSTM network to further synchronize spatiotemporal feature extraction from the residual network’s output, ultimately achieving single-station pollutant concentration predictions through the output layer. However, CNN-LSTM models face two challenges. First, converting irregularly distributed multi-station data within a region into one-dimensional or two-dimensional tensors for spatial feature extraction can distort the original spatial information distribution. Second, capturing the global evolution patterns and causal relationships of time series data is difficult, and the periodicity and trend similarities of pollutants are often overlooked, resulting in insufficient final prediction accuracy.

The limitations of combining CNNs and RNNs have become increasingly evident to researchers. Recent studies have investigated using GNNs, including GCNs and GATs, to extract spatiotemporal dependencies from multi-station data, thereby enhancing prediction performance [34, 35]. A notable combination method is the integration of Graph Neural Networks with Recurrent Neural Networks (GNN-RNN). For example, Han *et al*. proposed a Correlation Graph Attention-based Long Short-Term Memory network (CGA-LSTM) [35], a nested network that embeds the correlation attention mechanism within the graph attention mechanism to enhance spatiotemporal correlations. Liu *et al*. proposed a novel Spatiotemporal Adaptive Attention Graph Convolutional Model (STAA-GCN) [36] for city-level air quality prediction, prioritizing short-term PM2.5 concentration forecasts. STAA-GCN encodes multiple spatiotemporal dependencies and employs station-level attention to construct comprehensive spatiotemporal interactions between stations. Hu *et al*. introduced an Adaptive Hierarchical Graph Convolutional Neural Network (AHGCNN) [37], featuring an adaptive hierarchical graph convolutional structure that dynamically extracts multi-level spatial dependencies. This model integrates the unique topological structures of graph neural networks at different levels and incorporates an Adaptive Hierarchical Graph Convolutional Gated Recurrent Unit (AHGC-GRU) to capture the spatiotemporal dependencies of air quality data. Wang *et al*. proposed a spatiotemporal prediction framework, GC-SRTCN-L [38], which integrates graph convolution, temporal convolution, and linear modules. The synergistic effect of these three modules enables GC-SRTCN-L to effectively capture the spatiotemporal characteristics of PM2.5. Studies have demonstrated that GNN architectures perform exceptionally well in pollutant prediction tasks, providing technical inspiration and research ideas for this study.

Current research has propelled GNNs to the forefront of study. However, existing research on pollutant concentration prediction using GNNs must address three main challenges: (1) Reducing reliance on prior knowledge. Excessive dependence on prior knowledge can undermine the accuracy of spatial feature extraction, such as establishing spatial relationships between monitoring stations based on distance. GCN and AdapGCN networks are limited to achieving dynamic spatial correlation modeling. (2) Accounting for the dynamic evolutionary causal relationships in temporal data and the periodicity and similarity of pollutants. Existing studies on time correlation extraction often overly focus on techniques like recurrent networks or Transformers, neglecting the inherent properties of temporal data. For instance, a typical causal relationship is increased pollutant concentration during the morning peak due to pre-peak travel volume. (3) Addressing cumulative prediction errors. During the decoding phase of prediction models, dynamic decoding can cause errors to propagate dynamically across both temporal and spatial dimensions, leading to low prediction accuracy and high model inference costs. In response to these challenges, this paper further investigates multi-station pollutant concentration prediction.

## 3 Problem description

This study models the pollutant concentration monitoring network as a weighted undirected graph G=(V,E,A). Here, V denotes the set of stations in the monitoring network (with the total number of stations N=|V|); *E* represents the edges, which signify the relationships between stations; and $\text{A}\in {\text{R}}^{N\times N}$ is the weighted adjacency matrix, where ${\text{A}}_{v_{i},\;v_{j}}$ indicates the correlation between monitoring stations $v_{i}$ and $v_{j}$, defining the numerical relationship on the edge. At time step *t*, the pollutant concentrations over graph G are encoded as the graph signal ${\text{X}}_{t}\in {\text{R}}^{N\times C}$, where *C* denotes the number of pollutant attributes of interest (e.g., PM2.5, PM10). This study utilizes historical observations, denoted as $\text{X}=\left({\text{X}}_{t_{1}},{\text{X}}_{t_{2}},\ldots ,{\text{X}}_{t_{P}}\right)\in {\text{R}}^{P\times N\times C}$, collected from *N* monitoring stations over *P* time steps to forecast the target pollutant concentrations for *Q* future time steps, represented as $\hat{\text{Y}}=\left({\hat{\text{Y}}}_{t_{P+1}},{\hat{\text{Y}}}_{t_{P+2}},\ldots ,{\hat{\text{Y}}}_{t_{P+Q}}\right)\in {\text{R}}^{Q\times N\times 1}$.

## 4 Proposed approach

### 4.1 Framework overview

**Fig 1:**
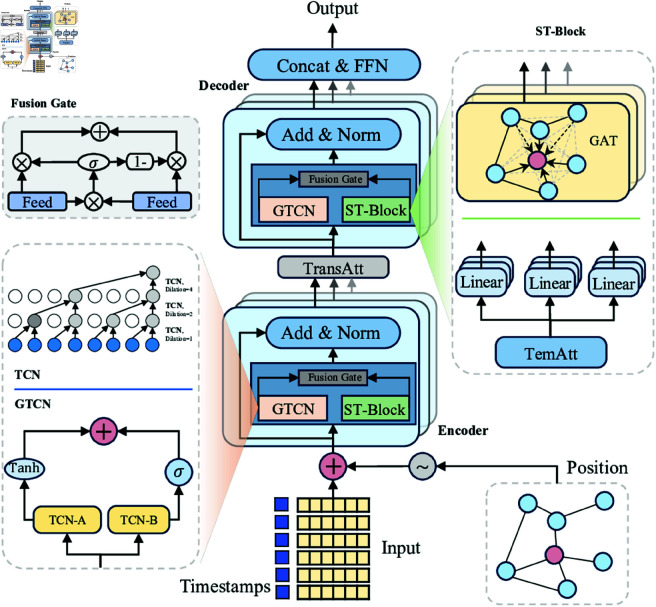
Overview of the STGATN model architecture. The model input consists of pollutant concentration, location, and timestamp data. The GTCN models temporal causal relationships at monitoring stations in a non-recursive manner. The ST-Block employs temporal attention to capture nonlinear correlations across time steps and integrates graph attention to model dynamic spatial dependencies. A fusion gate integrates temporal causal correlations with dynamic spatiotemporal features. A transformer attention layer between the encoder and decoder generates future representations.

Fig 1 shows the proposed STGATN framework, which adopts an encoder-decoder architecture. The encoder and decoder primarily comprise spatiotemporal embedding mechanism, ST-Block, GTCN, and fusion gate, which collectively extract spatiotemporal features from source inputs. Specifically, the proposed model integrates graph structure and temporal information within the spatiotemporal feature extraction process using the spatiotemporal embedding (STE) mechanism. The GTCN utilizes parallel dilation-based causal convolutions to extract temporal causal features while employing a gate unit to selectively transmit information, thereby enhancing the model’s expressiveness and performance. The ST-Block captures global dynamic dependencies through temporal attention and dynamically models spatiotemporal correlations using a graph attention network. The fusion gate conducts a weighted integration of temporal causal features and spatiotemporal correlation features. Additionally, a TransAtt structure is introduced between the encoder and decoder to transfer the encoder’s output features, mitigating error propagation in dynamic decoding. Notably, to streamline model design and minimize computational costs, all layers generate features with a uniform dimension of 32.

### 4.2 Spatiotemporal embedding mechanism

Embedding denotes the transformation of spatial and temporal attributes, such as the geographical locations of monitoring stations and temporal indicators, into learnable low-dimensional vector representations. Temporal information encapsulates the dynamic fluctuations and periodic regularities of pollutant concentrations, whereas the spatial positions of monitoring stations reflect distributional attributes and geographical correlations, thereby facilitating the modeling of topographic influences and spatial dependencies in pollutant diffusion. To address these factors, this study introduces a spatial position embedding mechanism that encodes each vertex as an updatable, learnable vector [39]. In addition, a temporal embedding mechanism is proposed to encode timestamps and capture temporal dynamics. Specifically, assuming a day consists of *T* discrete time steps, one-hot encoding is used to represent the day-of-week and hour-of-day for each time step as $\text{X}W\in {\text{R}}^{7}$ and $\text{X}D\in {\text{R}}^{T}$, respectively. These are then concatenated to form a composite vector $\text{X}F\in {\text{R}}^{7+T}$ [14]. Subsequently, a two-layer one-dimensional convolutional neural network (1-D CNN) is applied to convert the time vector into an embedding representation $\text{X}T\in {\text{R}}^{D}$. The model inputs historical *P* and future *Q* time steps, where the representation of time step *t*_*j*_ is $e_{t_{j}}\in {\text{R}}^{D}$, with $t_{j}=t_{1},...,t_{P},...,t_{P+Q}$. Similarly, the station position embedding can be represented as $e_{v_{i}}\in {\text{R}}^{D}$, where $v_{i}\in \text{V}$.

To acquire dynamic representations of monitoring stations, this study combines positional and timestamp representations into a unified spatiotemporal embedding (STE), as illustrated in Fig 1. For vertex $v_{i}$ at time step *t*_*j*_, the spatiotemporal embedding is defined as $e_{v_{i},t_{j}}=e_{t_{j}}+e_{v_{i}}$. Consequently, the spatiotemporal embeddings for *N* monitoring stations over *P* + *Q* time steps are represented as $\text{S}TE\in {\text{R}}^{\left(P+Q\right)\times N\times D}$. STE incorporates both spatial and temporal information and will be utilized in the Encoder, TransAtt, and Decoder.

### 4.3 Spatio-temporal graph attention block (ST-Block)

As illustrated in Fig 1(c), the ST-Block comprises GAT, TemAtt, and a fusion gate. The input to the *l*-th layer in encoder (or decoder) is denoted as ${\text{H}}_{ST}^{\left(l-1\right)}\in {\text{R}}^{S\times N\times D}$, where the hidden state of monitoring station $v_{i}$ at time step *t*_*j*_ is represented as $hst_{v_{i},\;t_{j}}^{\left(l-1\right)}\in {\text{R}}^{D}$. The outputs of the GAT and TemAtt in the *l*-th layer are denoted as ${\text{H}}_{S}^{l}\in {\text{R}}^{S\times N\times D}$ and ${\text{H}}_{T}^{l}\in {\text{R}}^{S\times N\times D}$, respectively, with the hidden states of station $v_{i}$ at time step *t*_*j*_ represented as $hs_{v_{i},\;t_{j}}^{\left(l\right)}\in {\text{R}}^{D}$ and $ht_{v_{i},\;t_{j}}^{\left(l\right)}\in {\text{R}}^{D}$. Following the fusion gate, the output of the ${\text{l}}^{\text{th}}$ layer is represented as ${\text{H}}_{ST}^{\left(l\right)}\in {\text{R}}^{S\times N\times D}$. Assume the length of input time steps is *S*.

#### 4.3.1 Temporal attention (TemAtt).

TemAtt is an adaptive module that captures nonlinear temporal correlations by dynamically assigning attention weights to different historical time steps. The pollutant concentration at a monitoring station is influenced by its historical observations, with correlations varying nonlinearly over time. To model these dynamics, this paper introduces the TemAtt mechanism to adaptively capture nonlinear correlations across time steps. In addition, temporal correlations are also influenced by the historical concentration change patterns at various stations. For instance, the pollutant concentration at monitoring station A may follow a unique trend similar to historical concentration patterns observed during the same periods. Therefore, this paper incorporates both the target pollutant features and spatiotemporal embeddings to model temporal correlations between time steps. Specifically, the hidden states are concatenated with the spatiotemporal embeddings, and multi-head attention is employed to compute the attention values. The correlation between time steps *t*_*j*_ and *t* at monitoring station $v_{i}$ is defined by Eq 1:

$$\mu_{t_{j},t}^{\left(k\right)}=\frac{\left\langle f_{t,1}^{\left(k\right)}\left(h_{v_{i},t_{j}}^{\left(l-1\right)}\|e_{v_{i},t_{j}}\right),f_{t,2}^{\left(k\right)}\left(h_{v_{i},t}^{\left(l-1\right)}\|e_{v,t}\right)\right\rangle }{\sqrt{d}}$$ (1)

where ∥ denotes the concatenation operation, ⟨ ⟩ represents the inner product, and the dimension of $f_{t,1}^{\left(k\right)}\left(h_{v_{i},\;t_{j}}^{\left(l-1\right)}∥e_{v_{i},\;t_{j}}\right)$ is 2*D*. Subsequently, $\mu_{t_{j},t}^{\left(k\right)}$ is normalized using the *SoftMax* function, as defined in Eq 2:

$$\beta_{t_{j},v}^{\left(k\right)}=\frac{exp\left(\mu_{t_{j},t}^{\left(k\right)}\right)}{\sum_{r=1}^{S}exp\left(\mu_{t_{j},t_{r}}^{\left(k\right)}\right)}$$ (2)

where $\mu_{t_{j},t}^{\left(k\right)}$ denotes the attention score between time steps *t*_*j*_ and *t* in the *k*-th attention head, reflecting the relative importance of $v_{i}$ to *t*_*j*_. After computing the attention scores, the hidden state of monitoring station $v_{i}$ at time step *t*_*j*_ is updated according to Eq 3:

$$ht_{v_{i},\;t_{j}}^{\left(l\right)}={∥}_{k=1}^{K}\left\{\sum_{r=1}^{S}\beta_{t_{j},v}^{\left(k\right)}.{f_{t,3}^{\left(k\right)}(h}_{v_{i},\;t_{r}}^{\left(l-1\right)})\right\}$$ (3)

where $f_{t,1}^{\left(k\right)}$, $f_{t,2}^{\left(k\right)}$, and $f_{t,3}^{\left(k\right)}$ in the *k*-th attention head represent distinct nonlinear transformations [14], with last dimension divided as *d* = 2*D*/*K*. The TemAtt module receives ${\text{H}}_{ST}^{l-1}$ as input and generates ${\text{H}}_{T}^{l}$ as output.

#### 4.3.2 Graph attention (GAT).

GAT is an adaptive mechanism that dynamically models time-varying spatial dependencies within a monitoring station network by assigning distinct attention weights to each station at every time step [39]. Due to meteorological and other factors, pollutants exhibit spatial diffusion in real environments. Consequently, the pollutant concentration at a target monitoring station is influenced by other monitoring stations to varying degrees [33]. This influence is highly dynamic and changes over time. To effectively model dynamic spatial dependencies, this study proposes a GAT mechanism that adaptively captures time-varying correlations among monitoring stations. The key idea is to dynamically assign distinct weights to different monitoring stations at various time steps. Specifically, the model initially concatenates the hidden states with the corresponding spatiotemporal embeddings and subsequently employs the scaled dot-product mechanism to compute the correlation between vertices $v_{i}$ and *v*, as shown in Eqs 4 and (5):

$$s_{v_{i},v}^{\left(k\right)}=\frac{\left\langle f_{s,1}^{\left(k\right)}\left(h_{v_{i},t_{j}}^{\left(l-1\right)}\|e_{v_{i},t_{j}}\right),f_{s,2}^{\left(k\right)}\left(h_{v_{i},t_{j}}^{\left(l-1\right)}\|e_{v,t_{j}}\right)\right\rangle }{\sqrt{d}}$$ (4)

$$\alpha_{v_{i},v}^{\left(k\right)}=\frac{\exp\left(s_{v_{i},v}^{\left(k\right)}\right)}{\sum_{v_{r}\in \text{V}}\exp\left(s_{v_{i},v_{r}}^{\left(k\right)}\right)}$$ (5)

After obtaining the attention scores $\alpha_{v_{i},v}^{\left(k\right)}$ in the *k*-th head, the hidden states can be updated using Eq 6:

$$hs_{v_{i},\;t_{j}}^{\left(l\right)}={∥}_{k=1}^{K}\left\{\sum_{v\in \text{V}}\alpha_{v_{i},v}^{\left(k\right)}.{f_{s,3}^{\left(k\right)}(h}_{v_{i},\;t_{j}}^{\left(l-1\right)})\right\}$$ (6)

The graph attention module takes ${\text{H}}_{ST}^{l-1}$ as input and produces ${\text{H}}_{S}^{l}$ as output. Importantly, its output can be fused with the temporal attention output via a fusion gate.

#### 4.3.3 Fusion gate.

The fusion gate serves as an adaptive mechanism that dynamically controls the integration of diverse features [40]. In ST-Block, TemAtt and GAT capture distinct yet complementary characteristics: temporal correlations and dynamic spatial dependencies, respectively. To seamlessly integrate these complementary properties, this study introduces a fusion gate that adaptively synthesizes their information, as illustrated in Fig 1(c). Within the encoder and decoder, the outputs of the fusion gate are represented as ${\text{H}}_{T}^{l}$ and ${\text{H}}_{S}^{l}$, with shapes ${\text{R}}^{P\times N\times D}$ in the encoder and ${\text{R}}^{Q\times N\times D}$ in the decoder. The fusion of ${\text{H}}_{ST}^{l}$ and ${\text{H}}_{T}^{l}$ is defined by Eqs 7 and (8):

$${\text{H}}_{ST}^{l}=Z⊙{\text{H}}_{T}^{l}+(1-Z)⊙{\text{H}}_{S}^{l}$$ (7)

$$Z=\sigma \left({\text{H}}_{T}^{l}*{\text{W}}_{z,1}+{\text{H}}_{S}^{l}*{\text{W}}_{z,2}+b_{z}\right)$$ (8)

where W and *b* denote learnable parameters, *Z* represents the weighting matrix, and * signifies the convolution operation. The fusion gate adaptively controls the flow of temporal correlations and dynamic spatial dependencies for each monitoring station and time step.

### 4.4 Gated temporal convolutional network (GTCN)

TCN can achieve an exponentially expanding receptive field by increasing network depth, thereby effectively capturing the full progression of temporal data dynamics. It supports long-term sequence feature extraction, enables parallel computation, and alleviates the problem of gradient explosion. Pollutant concentrations exhibit dynamic temporal variations and demonstrate distinct causal relationships. For example, the PM2.5 concentration near a monitoring station may gradually increase before 11:00 AM, peak at 11:00 AM, and subsequently decline during the following hour. This property is defined as a causal relationship in this paper. Therefore, accurately modeling these causal relationships is critical for guiding pollutant concentration predictions both efficiently and precisely. In contrast to traditional RNN approaches, this study utilizes dilated causal convolutions within TCN [41] to non-recursively capture temporal causal dependencies at monitoring stations. Dilated causal convolution operations typically skip specific feature values at defined strides, following a predetermined pattern, as illustrated in Fig 1(b). Assuming the past *T* time steps of observations at a monitoring station are given as $x\in {\text{R}}^{T\times D}$ and the filter is $filter\in {\text{R}}^{M\times D}$, the dilated causal convolution operation of *x* and the filter at time step *t* can be expressed as shown in Eq 9:

$$x*filter\left(t\right)=\sum_{m=0}^{M-1}filter\left(k\right)x\left(t-d\times m\right)$$ (9)

where *d* represents the dilation factor, which captures temporal dependencies and causal relationships across time steps; *M* represents the filter size.

To extract significant information from temporal data, this paper employs a GTCN to distill causal features, removing redundant information that could affect predictions. Similar to LSTM, its primary mathematical principle involves gating units that score the input data to filter out low-scoring features. Assuming the input to the GTCN is $\text{X}\in {\text{R}}^{N\times T\times D}$, the information flow control process is represented as follows:

$$\text{H}=Tanh\left({\text{W}}_{1}*\text{X}+b_{1}\right)⊙\sigma \left({\text{W}}_{2}*\text{X}+b_{2}\right)$$ (10)

where ${\text{W}}_{1}$, ${\text{W}}_{2}$, *b*_1_, and *b*_2_ are learnable network weight parameters; ‘⊙’ denotes element-wise multiplication; *Tanh* is the hyperbolic tangent function; and σ is the Sigmoid function, which transforms the input into a weight value between 0 and 1 to weight the features output by the Tanh function. The entire GTCN at layer *l* takes input ${\text{H}}_{GT}^{l-1}$ and produces output ${\text{H}}_{GT}^{l}$.

### 4.5 Transformer attention (TransAtt)

TransAtt module directly captures dependencies between historical and future time steps by transforming encoded input features into predictive future representations. To mitigate error propagation across extended prediction horizons, a TransAtt layer is inserted between the encoder and decoder. This layer explicitly models the relationships between individual historical and future time steps, transforming encoded features into future representations for the decoder. For monitoring station $v_{i}$, the correlation between the prediction time step *t*_*j*_ ($t_{j}=t_{P+1},...,t_{P+Q}$) and the historical time step *t* ($t=t_{1},...,t_{P}$) is calculated using spatiotemporal embeddings, as shown in Eqs (11) and (12):

$$\lambda_{t_{j},t}^{\left(k\right)}=\frac{\left\langle f_{t,1}^{\left(k\right)}\left(e_{v_{i},t_{j}}\right),f_{t,2}^{\left(k\right)}\left(e_{v_{i},t}\right)\right\rangle }{\sqrt{d}}$$ (11)

$$\gamma_{v_{i},t_{j}}^{\left(k\right)}=\frac{exp\left(\lambda_{t_{j},t}^{\left(k\right)}\right)}{\sum_{r=1}^{P}exp\left(\lambda_{t_{j},t_{r}}^{\left(k\right)}\right)}$$ (12)

Using the attention values $\gamma_{v_{i},t_{j}}^{\left(k\right)}$, the encoded input features are adaptively selected from *P* historical time steps and transformed into the decoder, as defined in Eq (13):

$${hst}_{v_{i},t_{j}}={∥}_{k=1}^{K}\left\{\sum_{r=1}^{P}\gamma_{t_{j},t_{r}}^{\left(k\right)}.f_{t,3}^{\left(k\right)}\left({hts}_{v_{i},t_{r}}\right)\right\}$$ (13)

where the input to the TransAtt mechanism is denoted as ${\text{H}}_{ST}$, while the corresponding output is represented as ${\text{H}}_{ST}^{\prime }$.

### 4.6 Encoder-decoder

As shown in Fig 1(a), STGATN employs an encoder-decoder architecture. Initially, a one-dimensional convolutional layer transforms the historical observations $\text{X}\in {\text{R}}^{P\times N\times C}$ into ${\text{H}}^{\left(0\right)}\in \;{\text{R}}^{P\times N\times D}$. This ${\text{H}}^{\left(0\right)}$ is then fed into the encoder, which consists of *L* layers, producing an output ${\text{H}}_{ST}\in {\text{R}}^{P\times N\times D}$. Subsequently, a TransAtt layer converts the encoded features ${\text{H}}_{ST}$ into future sequence representations ${\text{H}}_{ST}^{\prime }\in {\text{R}}^{Q\times N\times D}$. The decoder, comprising *L* layers, generates the output ${\text{H}}_{ST}^{\prime \prime }\in {\text{R}}^{Q\times N\times D}$. Finally, a fully connected layer produces the *Q*-time step prediction $\hat{\text{Y}}\in {\text{R}}^{Q\times N\times 1}$.

### 4.7 Loss function

This section details the complete feature extraction algorithm of STGATN. STGATN can be trained end-to-end using backpropagation by minimizing the mean absolute error (MAE) between the predicted and actual values, as shown in Eq (14):

$$L(\theta )=\frac{1}{Q}\sum_{r=1}^{Q}\left|{\text{Y}}_{t_{r}}-{\hat{\text{Y}}}_{t_{r}}\right|+\frac{\lambda }{2}\|\theta {\|}^{2}$$ (14)

where λ is the regularization parameter, and θ represents all the learnable parameters in the STGATN model.

## 5 Experiments

### 5.1 Data description

In this study, an air pollution dataset from 12 monitoring stations in Beijing, China, is utilized to evaluate the proposed pollutant concentration prediction model. The dataset, collected by the Beijing Environmental Monitoring Center, spans from March 1, 2013, to February 28, 2017 [42]. It comprises hourly data, totaling 35,064 hours, and includes six air pollutants (PM2.5, PM10, O3, SO2, NO2, and CO) along with five meteorological variables (wind speed, rainfall, pressure, dew point, and temperature). Consistent with existing research, 50% of the data was used for training, 25% for validation, and the remaining 25% for testing. Importantly, this study focuses exclusively on PM2.5 as the input feature, disregarding other pollutants and meteorological variables. The spatial distribution of the 12 monitoring stations was described in a previous study [38], while Table 1 reports the statistical characteristics of PM2.5 concentrations at each station.

**Table 1:** Data distribution.

Station	Minimum	Mean	Maximum	Standard derivation	25%	50%	75%
Tiantan	3	81.8	821	80.68	22	58	113
Aotizhongxin	3	82.38	898	81.87	22	58	114
Changping	2	71.47	882	72.33	18	46	100
Dingling	3	65.95	881	72.1	14	41	94
Dongsi	3	86.01	737	86.13	23	61	119
Guanyuan	2	82.73	680	81.25	23	59	114
Gucheng	2	83.98	770	82.64	24	60	116
Huairou	2	69.28	762	70.74	17	47	98
Nongzhanguan	2	84.58	844	86.25	22	59	116
Shunyi	2	79.11	941	80.85	19	55	111
Wanliu	2	83.46	957	81.73	23	59	116
Wanshouxigong	3	84.82	999	85.85	23	60	116

According to Table 1, the distribution of PM2.5 values varies significantly, causing inconsistencies in the data distribution among the training, validation, and test sets. Extreme values in the validation and training sets can lead to errors during normalization and denormalization. However, the differences in mean and standard deviation distributions are relatively small. Therefore, this paper employs the Mean-Std method to normalize the input data.

### 5.2 Baseline model

To validate the performance and practical applicability of the proposed model, eight baseline models were selected for comparison. These models encompass statistical methods, traditional machine learning techniques, and deep learning approaches. The specific baseline models include:

**• ARIMA** is a widely used statistical model for time series analysis and forecasting, combining three components: autoregression (AR), differencing (I), and moving average (MA).

**• SVR** is a regression method based on the principle of structural risk minimization. It aims to find a hyperplane in multi-dimensional space that maximizes the distance from all training data points while controlling the model’s complexity and generalization ability.

**• LSTM** is a special type of recurrent neural network (RNN) designed to address the common issue of gradient vanishing in traditional RNNs when processing and predicting time series data. Its unique architecture, including memory cells, input gates, output gates, and forget gates, allows it to learn long-term dependencies.

**• TCN** effectively captures local patterns in time series data by leveraging the local receptive field characteristics of CNNs. Additionally, TCN offers superior parallel computing capabilities and can handle longer sequences [41].

**• T-GCN** combines Graph Convolutional Networks (GCNs) with deep learning models for time series analysis, specifically designed to process graph-structured data with temporal dependencies. By integrating the temporal information of nodes and the graph topology, T-GCN effectively captures both the temporal evolution features and spatial dependencies within the graph [21].

**• MTGNN** is a graph neural network (GNN) framework specifically designed for multivariate time series prediction. It aims to capture latent spatial dependencies among variables. The framework automatically extracts unidirectional relationships among variables through a graph learning module and integrates external knowledge, such as variable attributes [43].

**• AGCRN** is an Adaptive Graph Convolutional Recurrent Network designed to automatically capture fine-grained spatial and temporal correlations in traffic sequences through adaptive modules and recurrent networks. The Graph Convolutional Network (GCN) is enhanced by two adaptive modules: the Node Adaptive Parameter Learning (NAPL) module and the Data Adaptive Graph Generation (DAGG) module [22].

**• GC-SRTCN-L** is a novel spatiotemporal prediction framework for PM2.5, comprising a graph convolutional module, a temporal convolutional module, and a linear module. This framework leverages historical PM2.5 data and related features from multiple stations to predict future PM2.5 concentrations across multiple stations, rather than a single station [38].

### 5.3 Evaluation metrics

To effectively evaluate the performance of the prediction methods, we utilize three evaluation metrics: Root Mean Square Error (RMSE), Mean Absolute Error (MAE), and the Index of Agreement (IA), as shown in Eqs (15), (16), and (17). RMSE and MAE are standard regression metrics used to calculate the differences between the observed values (Y) and the predicted values ($\hat{\text{Y}}$). Notably, smaller values indicate more accurate predictions.

$$\text{R}MSE=\sqrt{\frac{1}{N}\sum_{i=1}^{N}{\left({\hat{\text{Y}}}_{i}-{\text{Y}}_{i}\right)}^{2}}$$ (15)

$$\text{M}AE=\frac{1}{N}\sum_{i=1}^{N}\left|{\hat{\text{Y}}}_{i}-{\text{Y}}_{i}\right|$$ (16)

$$\text{I}A=1-\frac{\sum_{i=1}^{N}{\left({\hat{\text{Y}}}_{i}-{\text{Y}}_{i}\right)}^{2}}{\sum_{i=1}^{N}{\left(\left|{\hat{\text{Y}}}_{i}-\bar{\text{Y}}\right|+\sum_{i=1}^{N}\left|{\text{Y}}_{i}-\bar{\text{Y}}\right|\right)}^{2}}$$ (17)

where, *N* represents the number of samples, and $\bar{\text{Y}}$ denotes the mean of the observed values.

### 5.4 Experimental settings

The model’s training methodology involves learning on the training set and updating the model parameters if the MAE metric on the validation set decreases, continuing until the training concludes. The STGATN method proposed in this paper utilizes the Adam optimizer for weight updates. The model’s initial learning rate is 0.001, with a batch size of 32 and a maximum of 50 epochs. Additionally, the model incorporates seven hyperparameters: the number of multi-head attention layers (*L*), the hidden feature dimension (*D*), the number of attention heads (M), the number of layers in the gated temporal convolutional network ($\ddot{L}$), the dilation factor (*d*), the kernel size (*K*), and the regularization factor (λ). In this study, *D* is set to 32 and *M* to 4, with fewer network layers used to reduce training costs, i.e., *L*=1. Additionally, λ is set to 0.001. For more detailed information on model weight parameters, refer to the author’s GitHub community page. Unlike existing research, this study employs a shorter historical time step (*P*=12) as input, while maintaining a consistent target prediction time step (*Q*=6). Specific hyperparameters for the model are shown in Table 2. In this study, the STGATN method was implemented using PyTorch. Model training and testing were conducted on a server with an NVIDIA Tesla V100S-PCIE-32GB GPU and 24 CPU cores. The code for the proposed STGATN method is open-source and available on GitHub (https://github.com/zouguojian/Pollutant-prediction/tree/main/Pollution_predic).

**Table 2:** Model hyperparameters.

Component	Network Layer	hyperparameters	Value	Output Dimension
Spatio-temporal Embedding	1D Convolution	Kernel Size	1	[32, 12, 12, 32]
		Number of Layers	2	
		Activation Function	Yes	
Gated Temporal Convolutional Network	2D Convolution	Kernel Size	[1, 2]	[32, 12/6, 12, 32]
		Number of Layers	2	
		Dilation Factor	3	
ST-Block	Graph Attention	Hidden Nodes	32	Encoder
		Number of Attention Heads	4	[32, 12, 12, 32]
	GTemporal Attention	Hidden Nodes	32	Decoder
		Number of Attention Heads	4	[32, 6, 12, 32]
Gating Unit	2D Convolution	Kernel Size	[1, 1]	[32, 12/6, 12, 32]
		Number of Layers	2	
		Activation Function	Yes	
BridgeTrans	Global Attention	Hidden Nodes	32	[32, 6, 12, 32]
		Number of Layers	1	
		Number of Attention Heads	4	
Prediction Layer	2D Convolution	Kernel Size	[1, 1]	[32, 6, 12, 1]
		Number of Layers	2	
		Activation Function	Yes	
-	-	Batch size	32	-
-	-	Dropout	0.3	-
-	-	Decay Rate	0.9	-
-	-	Learning Rate	0.001	-
-	-	Epoch	50	-
-	-	Optimization Method	Adam	-
-	-	*K*	32/None	-
-	-	Number of Block Layers	1	-

### 5.5 Impact of adding pollutants on model performance

This study systematically evaluates the impact of incorporating additional pollutants (PM10, SO2, NO2, CO, and O3) into a PM2.5 prediction model using three key metrics: RMSE, MAE, and IA. As shown in Table 3, the results indicate a consistent decline in model performance upon the inclusion of any additional pollutant, with NO2 and SO2 causing the most significant adverse effects.

**Table 3:** The effectiveness of different pollutants for long-term PM2.5 concentration prediction.

Horizon	Metric	PM2.5+PM10	PM2.5+SO2	PM2.5+NO2	PM2.5+CO	PM2.5+O3	PM2.5
+1h	RMSE	19.210	21.141	21.321	20.690	19.966	**18.718**
	MAE	10.005	10.955	11.189	10.477	10.775	**9.848**
	IA	0.9862	0.9815	0.9811	0.9842	0.9842	**0.9865**
+2h	RMSE	23.675	25.110	25.719	24.973	24.148	**23.093**
	MAE	12.379	13.243	13.342	12.760	12.942	**12.181**
	IA	0.9787	0.9737	0.9719	0.9768	0.9768	**0.9791**
+3h	RMSE	27.366	28.570	29.459	28.525	27.703	**26.604**
	MAE	14.442	15.258	15.287	14.732	14.865	**14.172**
	IA	0.9711	0.9656	0.9625	0.9694	0.9691	**0.9717**
+4h	RMSE	30.521	31.624	32.896	31.620	30.877	**29.660**
	MAE	16.277	17.056	17.112	16.512	16.642	**15.951**
	IA	0.9636	0.9574	0.9524	0.9621	0.9612	**0.9643**
+5h	RMSE	33.358	34.408	35.934	34.389	33.821	**32.415**
	MAE	17.977	18.714	18.799	18.158	18.329	**17.606**
	IA	0.9560	0.9490	0.9424	0.9548	0.9530	**0.9567**
+6h	RMSE	35.956	36.986	38.566	36.974	36.517	**34.946**
	MAE	19.564	20.252	20.351	19.708	19.928	**19.160**
	IA	0.9482	0.9404	0.9327	0.9473	0.9446	**0.9490**

**Degradation in error metrics.** Across all forecasting horizons (1–6 hours), the inclusion of additional pollutants consistently leads to increased prediction errors. For example, for the horizon one prediction, the RMSE for PM2.5 alone is 18.718, whereas the combination of PM2.5 and NO2 increases the RMSE to 21.321, representing a 13.906% rise. Likewise, the MAE rises from 9.848 for PM2.5 alone to 11.189 for PM2.5+NO2, an increase of 13.617%. For the next six-step forecasting, the RMSE gap widens further, with PM2.5+NO2 reaching 38.566, compared to 34.946 for PM2.5 alone—an increase of 10.359%. These findings suggest that the inclusion of pollutants such as NO2 may introduce noise or conflicting signals, thereby impairing the model’s ability to accurately capture the underlying dynamics of PM2.5.

**Decline in model consistency (IA).** The IA consistently declines across all pollutant combinations, indicating reduced alignment between predicted and observed PM2.5 values. For example, for the horizon one prediction, the IA for PM2.5+NO2 drops from 0.9865 (PM2.5 alone) to 0.9811, a decrease of 0.547%. For the next six-step forecasting, the IA for PM2.5+SO2 declines from 0.9490 to 0.9404 (-0.906%). This downward trend suggests that multi-pollutant inputs may obscure PM2.5-specific patterns, potentially due to overfitting on irrelevant variables or unresolved inter-pollutant interactions.

**Pollutant-specific performance variations.** The PM2.5+PM10 combination shows the smallest performance degradation compared to PM2.5 alone (e.g., next six-step RMSE: 35.956 vs. 34.946; +2.890%). This modest degradation is likely due to the physical correlation between PM10 and PM2.5, as both are particulate pollutants. However, PM10 contributes no substantial predictive value, indicating redundancy in its inclusion. In contrast, the PM2.5+NO2 combination performs the worst, with increases in both RMSE and MAE exceeding 10% across all time horizons. As a gaseous pollutant with distinct spatiotemporal dynamics, NO2 likely introduces conflicting signals that interfere with the model’s ability to capture PM2.5 patterns.

### 5.6 Impact of adding meteorological factors on model performance

The integration of meteorological variables, temperature (TEMP), pressure (PRES), dew point (DEWP), rainfall (RAIN), and wind speed (WSPM), into the PM2.5 prediction model has nuanced effects on performance across long-term forecasting, as shown in Table 4. Although all variable combinations lead to increased prediction errors relative to the PM2.5-only baseline, the extent of performance degradation varies depending on the specific variable and forecast horizon, thereby highlighting the potential for targeted optimization strategies.

**Table 4:** The effectiveness of different meteorological factors for long-term PM2.5 concentration prediction.

Horizon	Metric	PM2.5+TEMP	PM2.5+PRES	PM2.5+DEWP	PM2.5+RAIN	PM2.5+WSPM	PM2.5
+1h	RMSE	20.859	19.700	20.330	20.059	19.915	**18.718**
	MAE	10.418	10.469	10.312	10.614	10.602	**9.848**
	IA	0.9842	0.9849	0.9833	0.9838	0.9835	**0.9865**
+2h	RMSE	25.037	24.140	24.622	24.252	23.816	**23.093**
	MAE	12.697	12.804	12.614	12.894	12.756	**12.181**
	IA	0.9769	0.9771	0.9751	0.9760	0.9762	**0.9791**
+3h	RMSE	28.336	28.097	28.235	27.793	27.205	**26.604**
	MAE	14.609	14.874	14.640	14.863	14.676	**14.172**
	IA	0.9700	0.9686	0.9668	0.9682	0.9687	**0.9717**
+4h	RMSE	31.293	31.654	31.357	30.989	30.199	**29.660**
	MAE	16.326	16.742	16.444	16.649	16.422	**15.951**
	IA	0.9630	0.9596	0.9586	0.9600	0.9611	**0.9643**
+5h	RMSE	33.979	34.763	34.192	33.898	32.940	**32.415**
	MAE	17.910	18.430	18.121	18.311	18.049	**17.606**
	IA	0.9559	0.9506	0.9501	0.9517	0.9532	**0.9567**
+6h	RMSE	36.402	37.485	36.778	36.550	35.448	**34.946**
	MAE	19.379	19.976	19.697	19.865	19.571	**19.160**
	IA	0.9488	0.9419	0.9415	0.9433	0.9453	**0.9490**

**Short-term prediction.** TEMP and DEWP contribute the largest increases in prediction error. For the horizon one forecast, combining PM2.5 with TEMP yields an RMSE of 20.859, an 11.438% increase compared to 18.718 for PM2.5 alone, and an MAE of 10.418 (+5.788%), likely reflecting temperature-related phenomena such as thermal inversions and hygroscopic particle growth. Similarly, the combination of PM2.5 and DEWP results in an RMSE of 20.330 (+8.612%) and an MAE of 10.312 (+4.712%), underscoring the influence of atmospheric humidity in modulating PM2.5 concentrations. In contrast, PRES and WSPM exhibit comparatively smaller effects on predictive accuracy. PM2.5 combined with PRES produces an RMSE of 19.700 (+5.246%) and an MAE of 10.469 (+6.306%), while the PM2.5+WSPM pairing leads to an RMSE of 19.915 (+6.395%) and an MAE of 10.602 (+7.656%), suggesting that wind speed may partially mitigate prediction error through its direct role in pollutant dispersion. RAIN introduces a moderate degradation in performance, with an RMSE of 20.059 (+7.164%) and an MAE of 10.614 (+7.779%), potentially due to transient scavenging effects that are difficult to capture over short temporal intervals.

**Long-term prediction.** Error escalation becomes more pronounced over extended forecasting horizons, particularly when incorporating PRES and RAIN. For the next six-step forecast, integrating PM2.5 with PRES yields an RMSE of 37.485 (+7.265%), an MAE of 19.976 (+4.259%), and an IA of 0.9419 (-0.748%), potentially reflecting the cumulative impact of atmospheric pressure on vertical atmospheric stability. The PM2.5+RAIN combination results in an RMSE of 36.550 (+4.590%), an MAE of 19.865 (+3.680%), and an IA of 0.9433 (-0.601%), suggesting the presence of unresolved interactions between precipitation events and PM2.5 resuspension or washout processes. Among all meteorological variables considered, WSPM exhibits the smallest degradation in predictive accuracy over longer horizons, with an RMSE of 35.448 (+1.437%), an MAE of 19.571 (+2.145%), and an IA of 0.9453 (-0.390%), highlighting its relative robustness and effectiveness in capturing advection-driven PM2.5 transport dynamics.

### 5.7 Performance comparison of different models

To emphasize the advantages of the proposed prediction method, we compared it with baseline models across different prediction horizons. The results, presented in Table 5, reveal several key findings from this study:

**Table 5:** Comparison of the STGATN model and baseline model proposed in this article for long-term pollutant concentration prediction tasks.

Horizon	Metric	ARIMA*	SVR*	LSTM*	TCN*	T-GCN*	MTGNN*	AGCRN*	GC-SRTCN-L*	STGATN
+1h	RMSE	22.158	21.584	22.278	24.99	19.325	19.006	19.099	**18.443**	18.718
	MAE	11.289	12.339	13.291	14.429	10.240	10.545	10.206	10.194	**9.848**
	IA	0.9820	0.9806	0.9805	0.9752	0.9856	0.9863	0.9858	**0.9871**	0.9865
+2h	RMSE	28.557	26.411	27.068	28.883	24.424	23.767	23.892	**22.976**	23.093
	MAE	14.407	15.425	16.222	16.921	13.036	13.350	12.921	12.901	**12.181**
	IA	0.9702	0.9711	0.9702	0.9663	0.9763	0.9783	0.9771	**0.9794**	0.9791
+3h	RMSE	34.039	32.687	30.219	31.081	28.685	27.845	27.843	**26.589**	26.604
	MAE	17.175	19.062	18.084	18.546	15.556	15.697	15.358	15.164	**14.172**
	IA	0.9577	0.9598	0.9619	0.9601	0.9664	0.9697	0.9682	**0.9721**	0.9717
+4h	RMSE	38.884	36.299	32.747	34.86	32.384	31.285	31.377	29.76	**29.660**
	MAE	21.667	22.016	19.492	21.464	17.890	17.555	17.231	17.076	**15.951**
	IA	0.9448	0.9404	0.9541	0.9496	0.9560	0.9601	0.9584	0.9641	**0.9643**
+5h	RMSE	43.218	37.396	36.588	36.693	35.613	33.964	34.472	32.518	**32.415**
	MAE	25.946	23.826	21.722	22.447	20.013	19.272	19.280	18.895	**17.606**
	IA	0.9319	0.9369	0.9404	0.9429	0.9453	0.9517	0.9484	0.9566	**0.9567**
+6h	RMSE	47.163	40.089	38.404	39.045	38.455	36.908	37.229	35.145	**34.946**
	MAE	27.032	25.317	23.188	24.140	21.965	21.059	21.106	20.599	**19.160**
	IA	0.9190	0.9235	0.9337	0.9341	0.9346	0.9420	0.9382	0.9479	**0.9490**

Table 5 clearly demonstrates the performance disparity between traditional models and deep learning approaches in air pollution forecasting. Specifically, statistical models such as ARIMA and classical machine learning methods like SVR exhibit markedly inferior predictive accuracy compared to deep neural networks. In the six-step PM2.5 forecasting task, ARIMA and SVR increased RMSE by 22.808% and 4.388%, respectively, relative to the LSTM model. Corresponding increases in MAE were 16.578% and 9.181%, while IA decreased by 1.574% and 1.092%. These performance gaps highlight the limited capacity of traditional methods to capture complex temporal dependencies and nonlinear dynamics inherent in air pollutant evolution. In addition, these findings emphasize the critical role of effective temporal feature extraction in improving predictive accuracy, particularly for long-term forecasting tasks. Deep learning models, especially those incorporating advanced temporal architectures, exhibit a pronounced advantage in modeling time-dependent patterns and adaptive relationships across multiple forecast horizons. Consequently, the results further substantiate the necessity of employing deep temporal learning frameworks for robust and reliable pollutant concentration prediction.

As shown in Table 5, TCN consistently outperforms LSTM network across various forecasting horizons. Specifically, in the six-step ahead PM2.5 prediction task, TCN reduces RMSE and MAE by 1.669% and 4.106%, respectively, and increases IA by 0.043%, relative to LSTM. These results underscore the superior capacity of TCN to capture nonlinear temporal dependencies, highlighting its advantages over recurrent architectures in modeling temporal dynamics. Nevertheless, PM2.5 concentration prediction is inherently a spatiotemporal task, as pollutant levels at a target station are significantly influenced by emissions and dispersion processes from neighboring locations. Hence, it is essential to incorporate spatial dependencies into the modeling framework to fully capture the diffusion-driven interactions among monitoring stations. As further demonstrated in Table 5, spatiotemporal models leveraging GNNs consistently surpass TCN in predictive performance. For the same forecasting task, models such as T-GCN, MTGNN, AGCRN, and GC-SRTCN-L achieve RMSE improvements of 1.511%, 5.473%, 4.651%, and 9.988%; MAE reductions of 9.010%, 12.763%, 12.568%, and 14.669%; and IA enhancements of 0.054%, 0.846%, 0.439%, and 1.477%, respectively. Furthermore, GC-SRACN-L outperforms T-GCN, demonstrating that a TCN combined with a graph neural network is more effective for modeling temporal correlations than an RNN combined with a GNN in spatiotemporal prediction tasks. These findings validate the critical role of spatiotemporal modeling in pollution forecasting and emphasize the enhanced predictive power gained through the integration of graph-based spatial representations with advanced temporal learning mechanisms. The results provide strong empirical support for the design of hybrid models in this study and offer practical implications for multi-station air pollution forecasting.

This study advances existing GNN-based spatiotemporal prediction frameworks by addressing three critical challenges: (1) eliminating reliance on predefined prior knowledge, (2) incorporating dynamic causal dependencies in temporal sequences, alongside the periodicity and inter-pollutant similarity patterns, and (3) alleviating cumulative error propagation in multi-step forecasting tasks. As reported in Table 5, the proposed STGATN model consistently outperforms all baseline models across multiple evaluation metrics. In the six-step ahead PM2.5 forecasting task, STGATN reduces RMSE by 25.904%, 12.829%, 9.004%, 10.498%, 9.125%, 5.316%, 6.132%, and 0.566% compared to ARIMA, SVR, LSTM, TCN, T-GCN, MTGNN, AGCRN, and GC-SRTCN-L, respectively. Correspondingly, it lowers MAE by 29.121%, 24.320%, 17.371%, 20.630%, 12.770%, 9.018%, 9.220%, and 6.986%, while improving IA by 3.264%, 2.761%, 1.639%, 1.595%, 1.541%, 0.743%, 1.151%, and 0.116%. Moreover, the advantages of STGATN become increasingly evident at longer forecasting horizons, demonstrating its strong generalization capability in long-term spatiotemporal pollutant prediction. These findings underscore the superior effectiveness of STGATN in capturing complex spatiotemporal dependencies and enhancing prediction accuracy across multiple monitoring stations.

Furthermore, we extended the task to long-term prediction, with the corresponding results summarized in Table 6. The proposed model exhibits robust performance across multiple prediction horizons, particularly excelling in short-term forecasting. At horizon one, the model attains an RMSE of 27.320, an MAE of 14.921, and an IA of 0.9701, highlighting its accuracy and strong agreement with observed values. Although long-term forecasts present challenges typical of extended prediction tasks, such as cumulative uncertainty and complex environmental variability, the model maintains a consistent trend in performance metrics. For example, for the next twenty-four-step forecasting, the RMSE and MAE values (71.837 and 44.243, respectively) remain within reasonable bounds for complex air quality forecasting, and the IA score (0.7309) continues to reflect meaningful predictive coherence. These findings underscore the model’s foundational reliability and its adaptability to temporal dynamics. Future improvements, such as incorporating memory-augmented architectures or hybrid physics-informed learning, could reduce error accumulation over longer horizons, further positioning the model as a robust framework for long-term environmental forecasting.

**Table 6:** The performance of long-term PM2.5 concentration prediction.

Metric	Horizon 1	Horizon 3	Horizon 6	Horizon 12	Horizon 24
RMSE	27.320	35.403	46.700	59.376	71.837
MAE	14.921	19.605	26.371	34.966	44.243
IA	0.9701	0.9472	0.9016	0.8284	0.7309

By minimizing uncertainties in air pollution forecasts, this study highlights that multi-step pollutant concentration prediction provides more direct practical benefits, enabling environmental management agencies to develop strategies earlier and advising the public to take preventive measures before outdoor activities. For example, policymakers can more accurately track pollution trends and implement proactive rather than reactive measures. In addition, local governments can leverage these predictions to impose temporary vehicle restrictions or optimize public transportation schedules during periods of high pollution.

### 5.8 Computation cost

As shown in Table 7, the proposed STGATN model exhibits notable advantages in parameter efficiency and computational resource utilization, as demonstrated by its performance on the Beijing dataset. A detailed analysis of its core strengths is provided below:

**Table 7:** Computing cost of the proposed method on the Beijing dataset.

Dataset	Model	Parameters	Training / (iteration = 100 rounds and batch size = 32)		Inference (batch size = 32)	
			Time cost	GPU usage	Time cost	GPU usage
Beijing	STGATN	85,827	17.563s	1,712MiB	37.028s	1,562MiB

**Parameter efficiency.** With only 85,827 parameters, STGATN constitutes a lightweight architecture relative to conventional deep learning models such as Transformers, which typically contain millions of parameters. Its compact design minimizes memory footprint and computational complexity, thereby supporting deployment on resource-constrained devices. A reduced parameter count also mitigates overfitting and accelerates both training and inference, which is particularly beneficial in scenarios requiring rapid adaptation to dynamic environmental conditions, such as real-time air quality forecasting.

**Training efficiency.** The model completes 100 training iterations in 17.563 seconds, with an average of 0.175 seconds per iteration. During training, GPU memory consumption remains stable at 1,712 MiB. This computational efficiency allows for the concurrent training of multiple models on a single GPU, thereby improving research scalability.

**Inference performance.** The model processes the entire test set in 37.028 seconds using a batch size of 32. Assuming a standard test set size (e.g., 8,748 samples), this corresponds to a per-sample inference time of 4.233 milliseconds. This low-latency performance satisfies real-time application demands, including instantaneous pollution alerts and deployment on mobile or embedded platforms. During inference, GPU memory consumption decreases to 1,562 MiB, indicating enhanced memory efficiency. This reduction is particularly important for edge computing platforms with constrained VRAM resources (e.g., IoT devices and unmanned aerial systems).

### 5.9 Case analysis

**Fig 2:**
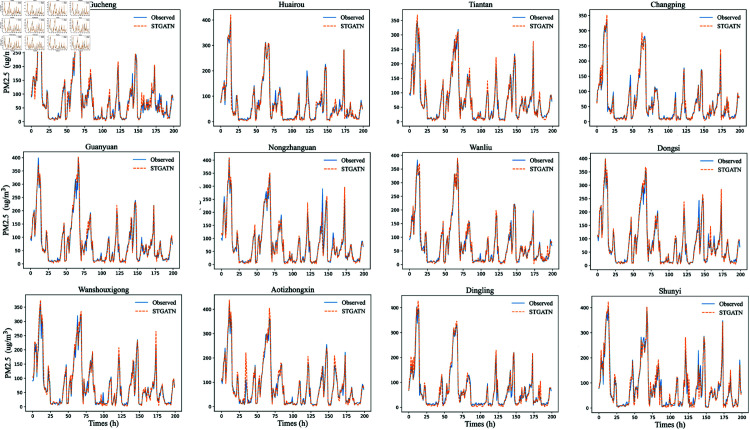
The prediction result of STGATN (prediction of the first time step). In the legend, the orange dashed line represents the predicted PM2.5 value, and the blue solid line represents the actual observed value. Randomly select 200 consecutive test samples from the test set.

**Fig 3:**
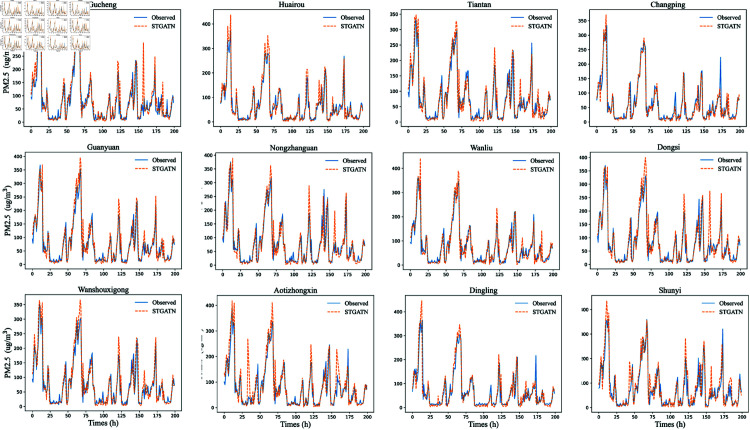
Prediction Results of STGATN (Third Time Step Prediction).

**Fig 4:**
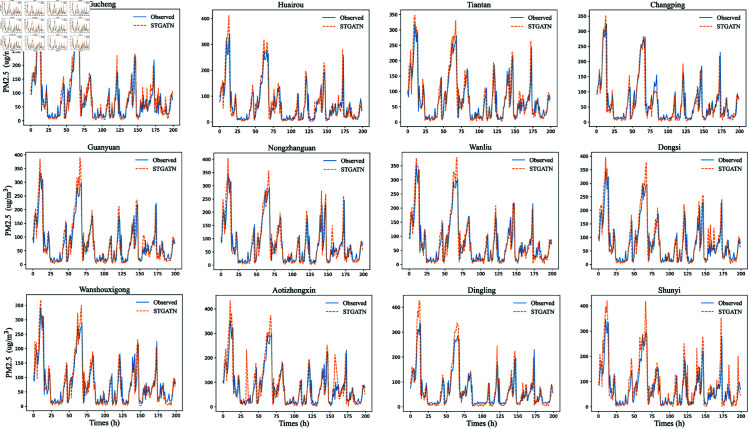
Prediction results of STGATN (6th time step prediction).

To provide a clearer demonstration of STGATN’s predictive performance, this section visualizes the PM2.5 predictions for twelve monitoring stations. The fitting results for time steps 1, 3, and 6 are shown in Figs 2, 3, and 4. From these visualizations, three notable findings emerge:

1. For PM2.5 concentration predictions across different time steps, STGATN demonstrates consistently accurate performance. Fig 2 illustrates that the model can precisely fit observed values for the next time step’s PM2.5 concentration. Even during periods of extreme pollutant concentration changes, such as between time steps 0-25 and 50-75, the model maintains its prediction accuracy. These accurate short-term predictions can address most travelers’ needs for health-conscious travel. For instance, travelers can implement temporary protective measures based on the forecasted air pollutant concentration for the next hour, thereby mitigating the health risks associated with inhaling high levels of airborne particulate matter. Consequently, effective multi-station PM2.5 concentration predictions can provide valuable advanced travel guidance for individuals in various regions.
2. As the prediction time step increases, predicting PM2.5 concentrations becomes more challenging. Figs 2, 3, and 4 illustrate that the model’s predictive performance for peak values gradually declines. However, this does not significantly affect the model’s overall performance. The figures reveal that when pollutant concentrations are below 300, the model maintains high prediction accuracy across different time steps. When pollutant concentrations exceed 300, the model exhibits minor deviations at extreme points, but the overall prediction trend remains highly consistent. This indicates that STGATN’s performance is influenced by the prediction horizon, consistent with the results in Table 5. Despite this, the overall prediction accuracy and trend estimation remain highly consistent with observations. These results highlight the practical application value of STGATN in real-life scenarios. For instance, a more precise prediction of pollutant spikes enables early warnings for at-risk populations, helping to minimize exposure to harmful air pollutants.
3. Finally, it is observed that in multi-station, multi-step PM2.5 concentration prediction tasks, the accuracy of predictions at some stations may be compromised. For instance, in Fig 3, the PM2.5 concentration predictions for the Changping and Dingling stations display reduced accuracy at time step 170. However, this issue does not appear in Fig 4. Based on these observations, this paper hypothesizes that in the process of synchronously optimizing predictions across multiple stations and time steps, the model may occasionally sacrifice the accuracy of certain time steps and stations to achieve overall optimal performance.

Accurately predicting PM2.5 concentrations is essential for effective air quality management and public health protection, given that PM2.5 dynamics are heavily influenced by both concentration levels and temporal variability. To rigorously evaluate the practical utility of the proposed STGATN model, this study conducts a systematic analysis of its performance across varying concentration levels (as shown in Table 8) and temporal patterns (as shown in Table 9), thereby elucidating both its strengths and limitations.

**Table 8:** The performance of the proposed STGATN on distinct concentration levels.

Horizon	Metric	0-50 $\mu g/m^{3}$	50-100 $\mu g/m^{3}$	100-200 $\mu g/m^{3}$	200-500 $\mu g/m^{3}$	500-800 $\mu g/m^{3}$	800-1000 $\mu g/m^{3}$
+1h	RMSE	**10.914**	14.177	21.735	39.353	98.402	275.086
	MAE	**5.701**	8.913	14.298	25.023	73.364	265.671
	IA	0.8597	0.8031	0.8702	**0.9210**	0.5268	0.0877
+2h	RMSE	**14.165**	17.547	26.588	47.862	118.454	330.919
	MAE	**6.990**	10.993	17.715	31.319	91.369	319.407
	IA	0.7889	0.7295	0.8174	**0.8860**	0.4451	0.0707
+3h	RMSE	**17.179**	20.181	30.001	54.319	137.611	377.916
	MAE	**8.260**	12.686	20.376	36.437	108.473	365.175
	IA	0.7233	0.6727	0.7795	**0.8549**	0.3853	0.0610
+4h	RMSE	**19.890**	22.448	32.824	59.857	155.280	419.467
	MAE	**9.488**	14.204	22.602	40.810	124.086	404.705
	IA	0.6664	0.6260	0.7482	**0.8262**	0.3431	0.0544
+5h	RMSE	**22.395**	24.439	35.247	64.702	173.066	459.525
	MAE	**10.710**	15.547	24.587	44.756	140.014	442.029
	IA	0.6170	0.5866	0.7215	**0.7997**	0.3091	0.0492
+6h	RMSE	**24.819**	26.184	37.361	68.948	190.893	497.758
	MAE	**11.926**	16.763	26.387	48.318	155.974	477.551
	IA	0.5721	0.5538	0.6987	**0.7755**	0.2816	0.0451

**Table 9:** The performance of the proposed STGATN on distinct temporal patterns.

Horizon	Metric	00:00-06:00	06:00-12:00	12:00-18:00	18:00-00:00
+1h	RMSE	**15.847**	19.868	17.605	18.420
	MAE	**8.573**	10.221	9.678	9.988
	IA	**0.9895**	0.9831	0.9879	0.9891
+2h	RMSE	**18.892**	25.334	21.660	25.255
	MAE	**10.149**	12.906	12.284	12.606
	IA	**0.9844**	0.9723	0.9819	0.9792
+3h	RMSE	**22.069**	29.027	25.161	29.641
	MAE	**11.819**	14.997	14.704	14.547
	IA	**0.9778**	0.9631	0.9758	0.9705
+4h	RMSE	**25.042**	32.247	28.241	32.843
	MAE	**13.505**	16.975	16.715	16.083
	IA	**0.9705**	0.9540	0.9698	0.9625
+5h	RMSE	**27.891**	34.619	31.459	35.447
	MAE	**15.239**	18.567	18.639	17.490
	IA	**0.9628**	0.9464	0.9627	0.9545
+6h	RMSE	**31.102**	36.506	34.542	37.594
	MAE	**17.024**	19.959	20.468	18.778
	IA	**0.9535**	0.9404	0.9549	0.9469

The STGATN model demonstrates strong performance in predicting PM2.5 concentrations under low to moderate pollution conditions, establishing it as a reliable tool for routine air quality monitoring. In the low-concentration range (0–50 $\mu g/m^{3}$), the model achieves high predictive accuracy, with a 1-hour RMSE of 10.914, MAE of 5.701, and IA of 0.8597, outperforming existing benchmarks in modeling steady-state pollution dynamics. For moderate concentrations (50–200 $\mu g/m^{3}$), the model retains robust predictive capability. Notably, in the 200–500 $\mu g/m^{3}$ range, it achieves a 1-hour IA of 0.9210, indicating its effectiveness in modeling transitional pollution scenarios. Even at extended forecast horizons, such as a 6-hour prediction in the 500–800 $\mu g/m^{3}$ range, the model maintains interpretable error metrics, with an RMSE of 68.948 and MAE of 48.318, supporting its applicability in operational forecasting. These results underscore the model’s precision in routine pollution environments, positioning it as a viable solution for urban air quality management and early public health warning systems.

The STGATN model demonstrates consistent performance across daily time segments, with particularly strong results during stable nighttime conditions (00:00–06:00). During this period, the model achieves peak performance, with a 1-hour RMSE of 15.847, MAE of 8.573, and IA of 0.9895, benefiting from stable meteorological conditions such as low wind speeds and thermal inversion. Even at 6-hour forecast horizons, nighttime predictions maintain high accuracy (RMSE = 31.102, MAE = 17.024, and IA = 0.9535), highlighting the model’s robustness under prolonged stable conditions. During dynamic daytime periods (e.g., 06:00–18:00), the model remains operationally reliable. For instance, between 12:00–18:00, it achieves a 3-hour RMSE of 25.161, MAE of 14.704, and IA of 0.9758, indicating effective adaptation to environmental variability influenced by human activity and solar radiation. This temporal adaptability supports continuous 24/7 air quality monitoring and positions the model as a practical tool for real-time decision-making in urban environments.

## 6 Conclusion

This paper addresses the challenge of multi-station pollutant concentration prediction by examining spatiotemporal pollutant diffusion and addressing issues related to pollutant variation patterns and prediction error propagation. Specifically, a spatiotemporal attention module with residual connections (ST-Block) is proposed as the vital component of the encoder and decoder. This module comprises a gated temporal convolution network, a spatiotemporal attention network, and a gated fusion unit. Specifically, the gated temporal convolution network utilizes dilated causal convolutions to extract causal temporal features from inputs and selectively passes these features through gated units to enhance causal relationship modeling. In addition, the spatiotemporal attention network operates without prior knowledge to comprehensively model global dynamic spatiotemporal correlations according to spatiotemporal embeddings and inputs. Moreover, the gated fusion unit performs a weighted fusion of the causal temporal features and spatiotemporal correlation features. Furthermore, a temporal transformer structure is incorporated between the encoder and decoder to learn the historical periodicity and similarity. This structure facilitates the conversion of encoder output features into the decoder while alleviating the prediction error propagation during the dynamic decoding process.

Experiments conducted on a real-world dataset with twelve monitoring stations demonstrate that STGATN achieves state-of-the-art results across RMSE, MAE, and IA metrics, highlighting its significant advantages in long-term multi-station pollutant concentration forecasting. The experiment further validates STGATN by visualizing randomly selected sample cases, showing that it accurately fits actual pollutant concentration and effectively adapts extreme values. The model maintains minimal prediction deviations while accurately forecasting future pollutant concentration trends. To align with existing prediction models, this study focuses on predicting PM2.5 concentration for the next six steps. Future research will extend the prediction horizon to 24 hours or one week to better address long-term pollutant concentration. If additional publicly available datasets from other geographic regions become accessible, we will integrate them to further assess the robustness and generalizability of the proposed model.
